# The release of host-derived antibodies bound to the variant surface glycoprotein (VSG) of *Trypanosoma brucei* cannot be explained by pH-dependent conformational changes of the VSG dimer

**DOI:** 10.12688/openreseurope.16783.1

**Published:** 2024-04-24

**Authors:** Patrick Eirich, Pavel Nesterov, Sergey Shityakov, Ekaterina V. Skorb, Bodo Sander, Jens Broscheit, Thomas Dandekar, Nicola G. Jones, Markus Engstler

**Affiliations:** 1 Department of Cell & Developmental Biology, Biocentre, University of Würzburg, Würzburg, Bavaria, 97074, Germany; 2 Department of Anaesthesiology, Intensive Care, Emergency and Pain Medicine, Würzburg University Hospital, University of Würzburg, Würzburg, Bavaria, 97080, Germany; 3 Infochemistry Scientific Center, Laboratory of Chemoinformatics, ITMO University, Saint Petersburg, Saint Petersburg, 191002, Russian Federation; 4 Department of Bioinformatics, Biocentre, University of Würzburg, Würzburg, Bavaria, 97074, Germany; 5 Rudolf Virchow Center for Experimental Biomedicine, University of Würzburg, Würzburg, Bavaria, 97080, Germany

**Keywords:** Trypanosoma brucei, variant surface glycoprotein, molecular dynamics, conformational shift, epitopes

## Abstract

**Background:**

*Trypanosoma brucei* is a protozoan parasite that evades the mammalian host’s adaptive immune response by antigenic variation of the highly immunogenic variant surface glycoprotein (VSG). VSGs form a dense surface coat that is constantly recycled through the endosomal system. Bound antibodies are separated in the endosome from the VSG and destroyed in the lysosome. For VSGs it has been hypothesized that pH-dependent structural changes of the VSG could occur in the more acidic environment of the endosome and hence, facilitate the separation of the antibody from the VSG.

**Methods:**

We used size exclusion chromatography, where molecules are separated according to their hydrodynamic radius to see if the VSG is present as a homodimer at both pH values. To gain information about the structural integrity of the protein we used circular dichroism spectroscopy by exposing the VSG in solution to a mixture of right- and left-circularly polarized light and analysing the absorbed UV spectra. Evaluation of protein stability and molecular dynamics simulations at different pH values was performed using different computational methods.

**Results:**

We show, for an A2-type VSG, that the dimer size is only slightly larger at pH 5.2 than at pH 7.4. Moreover, the dimer was marginally more stable at lower pH due to the higher affinity (ΔG = 353.37 kcal/mol) between the monomers. Due to the larger size, the predicted epitopes were more exposed to the solvent at low pH. Moderate conformational changes (ΔRMSD = 0.35 nm) in VSG were detected between the dimers at pH 5.2 and pH 7.4 in molecular dynamics simulations, and no significant differences in the protein secondary structure were observed by circular dichroism spectroscopy.

**Conclusions:**

Thus, the dissociation of anti-VSG-antibodies in endosomes cannot be explained by changes in pH.

## Introduction

African trypanosomiasis, also known as sleeping sickness, is a neglected tropical disease (NTD) defined by the World Health Organization (WHO). Over the past 120 years, this disease has experienced severe outbreaks that lasted for extended periods, with occasional temporary declines in cases. Currently, we are in a phase of low incidence, which has diverted the attention of the Global North. Urgent investment in basic research on NTDs is necessary to prepare for future outbreaks, particularly zoonotic diseases such as sleeping sickness. Apart from its impact on human health caused by *Trypanosoma brucei* subspecies, *Trypanosoma congolense* and *T. vivax* also infect livestock, causing a similar sickness called "nagana." The negative consequences of animal infections on human well-being are significant but poorly quantified, as rearing cattle in endemic areas becomes challenging ^1, 2^.

In contrast to many other eukaryotic parasites, such as *Plasmodium*, *Leishmania*, or *Toxoplasma*, which primarily reside intracellularly within their mammalian hosts, African trypanosomes thrive in the hostile environment of the mammalian bloodstream. Consequently, these flagellates are continuously targeted by the host's immune system, to which they have developed potent evasion strategies. Their primary defense mechanism involves changing their surface coat, which is composed of a variant surface glycoprotein (VSG), through stochastic activation of different VSG genes. This ability enables them to periodically present the host with novel antigenic cell surfaces ^3, 4^.

The variant surface glycoprotein (VSG) comprises over 95 % of all plasma membrane proteins and envelops the entire trypanosome cell ^5, 6^. These proteins serve as the main antigens on the cell surface, effectively shielding the invariant epitopes of the cell surface from the host immune system ^7^. VSGs form a family of glycoproteins that exhibit similar structures and are anchored to the cell surface by glycosyl-phosphatidyl-inositol (GPI) anchors ^8, 9^. Each VSG monomer has a molecular mass of 45–55 kDa and consists of two or three domains ^10^: the VSG N-terminal domain (350–400 residues) and one or two C-terminal domains (30–70 residues each). The N- and C-terminal domains are separated by a hinge region. Crystal structure analysis has revealed the structures of the N-terminal domains of different VSGs to fall into two broad classes, termed A and B which can be further grouped into subclasses ^11^. The N-terminal domains of all A-type VSGs appear to be homodimeric. Although there is typically less than 25% sequence similarity at the amino acid level among different VSGs, these proteins have similar overall shapes and VSGs belonging to the same classes/subclasses adopt similar folds. This VSG fold likely plays a crucial role in the protective function of the surface coat, while the variation in primary sequence enables antigenic variation to counteract the host's immune response ^12^. In contrast to the N-terminal domains, the C-terminal domains, sandwiched between the N-terminal domain and the plasma membrane, display a higher degree of primary sequence identity. The structures of three VSG C-terminal domains have been elucidated using nuclear magnetic resonance spectroscopy, and revealed a novel protein fold that is likely to be conserved among different C-terminal domains ^13, 14^.

In addition to antigenic variation, a process known as antibody clearance is believed to occur on the surface of trypanosomes. These parasites have developed a hydrodynamic defense mechanism to counteract the immune attack by the host. Due to their continuous and directional swimming, the trypanosome cell surface is constantly exposed to hydrodynamic flow. Host-produced antibodies attached to variant surface glycoproteins (VSG ^IgG^) are carried by this flow towards the posterior pole of the cell, where they undergo rapid endocytosis ^15^. The internalization of VSG ^IgG^ from the trypanosome cell surface involves three consecutive steps. Initially, VSG ^IgG^ accumulates at the posterior pole of the cell due to hydrodynamic drag forces. Next, VSG ^IgG^ enters the flagellar pocket through bulk membrane flow. The flagellar pocket is a small invagination of the plasma membrane and serves as the sole site for endocytosis and membrane recycling in *T. brucei*. Finally, VSG ^IgG^ is rapidly internalized through clathrin-mediated endocytosis. Within the endosomes, the antibodies are separated from VSG ^IgG^ and sorted through late endosomes to a single lysosome, where they are destroyed. In contrast, the VSG is efficiently directed to recycling endosomes via the flagellar pocket and subsequently returned to the cell surface. While it is established that hydrodynamic protein sorting and rapid plasma membrane recycling play a significant role in eliminating host-derived immunoglobulins from the trypanosome cell surface, there are still several unanswered questions. One of these questions is how the host antibody is released from the internalized VSG ^IgG^ complex. One possibility is that a major structural change occurs in the VSG dimer due to the more acidic environment of the endosome. In *T. brucei* endosomes the pH has suggested to vary between pH 5.2 to 5.5 ^16^.

In this study, we present the results of biochemical experiments and numerical simulations aimed at testing this hypothesis. We found only minimal conformational changes at acidic pH compared to normal blood pH. Consequently, our work appears to eliminate the most apparent mechanism for the separation of antibodies from internalized VSG ^IgG^ complexes.

## Methods

### Cultivation of *T. brucei*

The trypanosome cell line 13–90 ^17^, expressing the VSG MITat1.2 ^18^ were cultured at 37 °C and 5 % CO _2_ atmosphere with H _2_O saturation. Cell concentration was determined in regular intervals by manual counting. Cell densities above 1×10 ^6^ cells/ml were avoided by dilution with prewarmed HMI-9 medium ^19, 20^ to ensure the exponential growth phase. This was done using a HMI-9 selection medium containing 5 µg/ml hygromycin and 2.5 µg/ml geneticin (G418) and 10 % heat-inactivated fetal calf serum (Gibco, Catalog No. 15975161). When cells needed to be grown to higher density for VSG purification, trypanosomes were partitioned into conical flasks. To provide optimal environmental conditions and avoid sedimentation of parasites, cultures were incubated on an orbital shaker at 44 rpm and 37 °C with 5 % CO _2_ atmosphere and H _2_O saturation ^20^.

### Cell harvest

To purify soluble VSG (sVSG) in which the lipid part of the GPI-anchor has been removed by GPI-PLC activity, the original protocols of Cross were referred to ^21^. At a cell density of 2.45 to 4.07×10 ^6^ cells/ml, trypanosomes were harvested by centrifugation at 800 × *g* and 4 °C for 20 min. The supernatant was aspirated, and the cell pellets were then washed a total of three times with TDB ^20^ with centrifugation steps at 1500 × *g* and 4 °C for 10 min. After the last wash, the combined cell pellet was resuspended in 10 mM sodium phosphate buffer (pH 8.0) and 0.1 mM TLCK and proteinase inhibitor cocktail (1× Roche cOmplete EDTA free, SKU 11873580001) was added. The cell suspension was flash-frozen in liquid nitrogen and stored at -80 °C until protein purification. For a more detailed description of the cultivation and the cell harvest see the attached dataset ^20^.

### Protein purification by anion exchange chromatography

The frozen cell suspension was thawed, placed in a water bath at 37 °C for 5 min, and then centrifuged for 5 min at 4 °C and 10,000 × *g*. Cell pellets were resuspended in 10 mM sodium phosphate buffer (pH 8.0), 0.1 mM TLCK, and Roche proteinase inhibitor cocktail cOmplete EDTA-free ^20^. The sequence of heating in a water bath, centrifugation, and resuspension was performed a total of three times. The combined supernatants were centrifuged using an Amicon 10k Millipore centrifugal filter unit at 5000 × *g* and 4 °C for 25 min to reduce the volume. The buffer was then exchanged to 20 mM TRIS-HCl (pH 8.0) using the same filter unit. Using the ÄKTAprime plus and a HiTrapQ HP column, the sVSG was purified using the Anion Exchange HiTrap Q program. During the program, the NaCl gradient of the 20 mM TRIS-HCl (pH 8.0) running buffer changed from 0 to 1 M. The sVSG eluted at NaCl concentrations between 250 and 400 mM.

### Preparation for size-exclusion chromatography and circular dichroism measurements

For size-exclusion chromatography (SEC), the purified sVSG fraction was buffer exchanged to 50 mM HEPES, pH 7.4, with 150 mM NaCl using a 10 kDa cutoff Amicon filter unit. For circular dichroism (CD) measurements, fractions containing purified sVSG were pooled in the Amicon filter unit and concentrated at 5000 × *g* and 4 °C and buffer exchanged to 20 mM sodium phosphate buffer (pH 7.4). Some of the concentrate was removed and aliquots were prepared to conduct experiments on and the remainder was washed with 20 mM phosphate buffer (pH 5.2) several times (5000 × *g* and 4 °C) and aliquots were also prepared. All aliquots were stored in the -80 °C refrigerator until use in experiments. For a more detailed description of the protein purification and the preparation for the SEC and CD see the attached dataset ^20^.

### Size-exclusion chromatography

SEC of MITat1.2 at pH 7.4 was performed on an ÄKTA pure using a Superdex 200 Increase 10/300 GL column and 50 mM HEPES buffer (pH 7.4) with 150 mM NaCl as the running buffer. First, the sample concentration was determined using the Nanodrop 2000c by absorbance at 280 nm. The VSG sample was then mixed with HEPES buffer in the correct ratio so that a concentration of 5 mg/ml was achieved. A 100 µl sample was injected into the ÄKTA pure and the programme was started. The flow rate was 1 ml/min and the UV absorbance was measured at 260 and 280 nm.

For SEC of MITat1.2 at pH 5.2, 20 mM sodium acetate buffer (pH 5.2) with 150 mM NaCl was used as the running buffer. The sample was first buffered so that it was in 20 mM sodium acetate buffer (pH 5.2). The protein concentration was determined using the Nanodrop 2000c by absorbance at 280 nm. The VSG sample was then mixed in the correct proportion with sodium acetate buffer so that a concentration of 5 mg/ml was achieved. A 100 µl sample was injected into the ÄKTA pure and the programme was started. The flow rate was 1 ml/min and the UV absorbance was measured at 260 and 280 nm.

### Circular dichroism spectroscopy

CD spectra in the far UV range from 185 nm to 260 nm were recorded using a JASCO J-810 spectropolarimeter in a temperature-controlled quartz cuvette with a path length of 1 mm. Measurements were performed at a protein concentration of 5 µM in 20 mM phosphate buffer (pH 7.4 and pH 5.2) in 0.5 nm steps. The scan speed was 50 nm/min, and the bandwidth was 1.5 nm with a response time of 1 s. The measurement temperatures were 20 and 90 °C. In order to free the CD spectra from interfering signals during individual measurements, 25 spectra were recorded and artifacts were eliminated by averaging over time. The data were subsequently normalized for molar ellipticity. The decrease of the CD signal at 200 nm between 20 and 90 °C was observed using the JASCO J-810 spectropolarimeter and the PTC-423S temperature control system to obtain melting curves. The measurements were performed with a protein concentration of 5 µM in 20 mM phosphate buffer (pH 7.4 and pH 5.2) in a 1 mm quartz cuvette. The temperature increase was 1 °C/min.

To calculate the secondary structure elements of MITat1.2 at pH 7.4 and pH 5.2 the online server DichroWeb was used ^22^. Using the CDSSTR algorithm, the CD spectroscopy curves of MITat1.2 at pH 5.2 and pH 7.4 were compared to the SMP180 reference set ^23, 24^.

The protein secondary structure of optimized geometries at different pH was predicted using the MOE algorithm, which is based on an implementation of the Bayesian prediction formalism ^25^. The probability distributions were directly estimated from a non-redundant training set of solved protein structures.

### Protein stability prediction

For the evaluation of the VSG protein stability at different pH parameters, the Rosetta refinement protocol (RRID:SCR_015701) incorporating the Monte-Carlo (MC) approach, was used ^26^. The Rosetta software is known for its capabilities in protein structure prediction and refinement ^27^. The MC algorithm predicts the protein stability by matching the root-mean-square-deviation (RMSD) values with the energy scores measured in the Rosetta energy units (REU). The Boltzmann constant (kT) value of 0.8 was used as the "temperature" parameter to accept or reject moves based on the Metropolis criterion ^28^. The Metropolis criterion was used to determine whether a move to a new state (in this case, a protein conformation) is accepted or rejected based on the change in energy and temperature. To carry out the simulations, a "trajectory" number of 1000 was set according to the Metropolis criterion. This means that the algorithm explored 1000 different trajectories, and only the lowest-energy structures were outputted. This approach aligns with the standard protocol outlined by Wang *et al.* ^29^.

### Variant surface glycoprotein (VSG) geometry preparation

The intact VSG protein, MITat1.1, contains two domains: an N-terminal domain (crystal structure, PDB-ID: 5LY9) and a C terminal domain (NMR-structure, PDB-ID: 5M4T). A SAXS data-based model of the entire structure has previously been generated by fitting these high-resolution structures into the lower-resolution SAXS data ^30^.

The structure was refined using the Swiss-MODEL (RRID:SCR_013032) algorithm and MODELLER (RRID:SCR_008395) software ^31, 32^. The minimization and stereochemical validation of the 3D model was performed using the MOE software with the RMS gradient of 0.1 kcal/mol/A2 to remove steric clashes.

In order to simulate protein at different pH of the medium, two separate protonation states of VSG were obtained utilizing the PROPKA algorithm ^33, 34^. In order to predict epitopes of the VSG the Machine Learning algorithm EPIDOPE ^35^ was used.

### Molecular Dynamics simulations

Molecular dynamics simulations of VSG at different protonated states were performed utilizing the GROMACS (RRID:SCR_014565) program package ^36^. Interatomic interactions were described by the AMBER98SB force field ^37^ with topology and charges generated by GROMACS internal tools. For the water model, the rigid non-polarizable TIP3P ^38^ model was used. The cut-off for non-bonded and long-range interactions was 1.2 nm. For the calculation of long-range Coulomb interactions, the Particle-Mesh Ewald was used ^39^. Energy minimization was performed with the steepest descent algorithm. Simulations were performed in the NPT ensemble at T = 300 K and P = 1 atm in the cubic unit cell (approximately 174 × 174 × 174 A ^3^) for 200 ns each. The integration timestep for trajectory calculations was 2 fs.

### Analysis of obtained trajectories

The resulting trajectories of VSG were firstly analysed utilizing GROMACS internal tools to obtain Square Mean Root Deviations and Mean Root Deviations of protein motion concerning the first frame of the simulation, Root Mean Square Fluctuations, Solvent Accessible Surface Area of protein and epitopes, Gyration radius, Buried Surface Area, Molecular Volume and the number of hydrogen bonds for protein both intramolecular and between monomers. HawkDock server ^40^ was used in order to calculate the Gibbs free energy of assembly of two VSG monomers into the dimer. Principle component analysis was performed for VSG movements during the simulation utilizing the RStudio Bio3D (RRID:SCR_024266) package ^41^. Based on the Principal Component Analysis, the potential energy surfaces for protein at pH 5.2 and pH 7.4 were obtained.

## Results

### Size exclusion chromatography

Size exclusion chromatography was performed to determine if MITat1.2 was present as a homodimer at pH 5.2. If MITat1.2 were present as a monomer at pH 5.2, this would affect the CD measurement and the conclusions would be biased.

**Figure 1.**
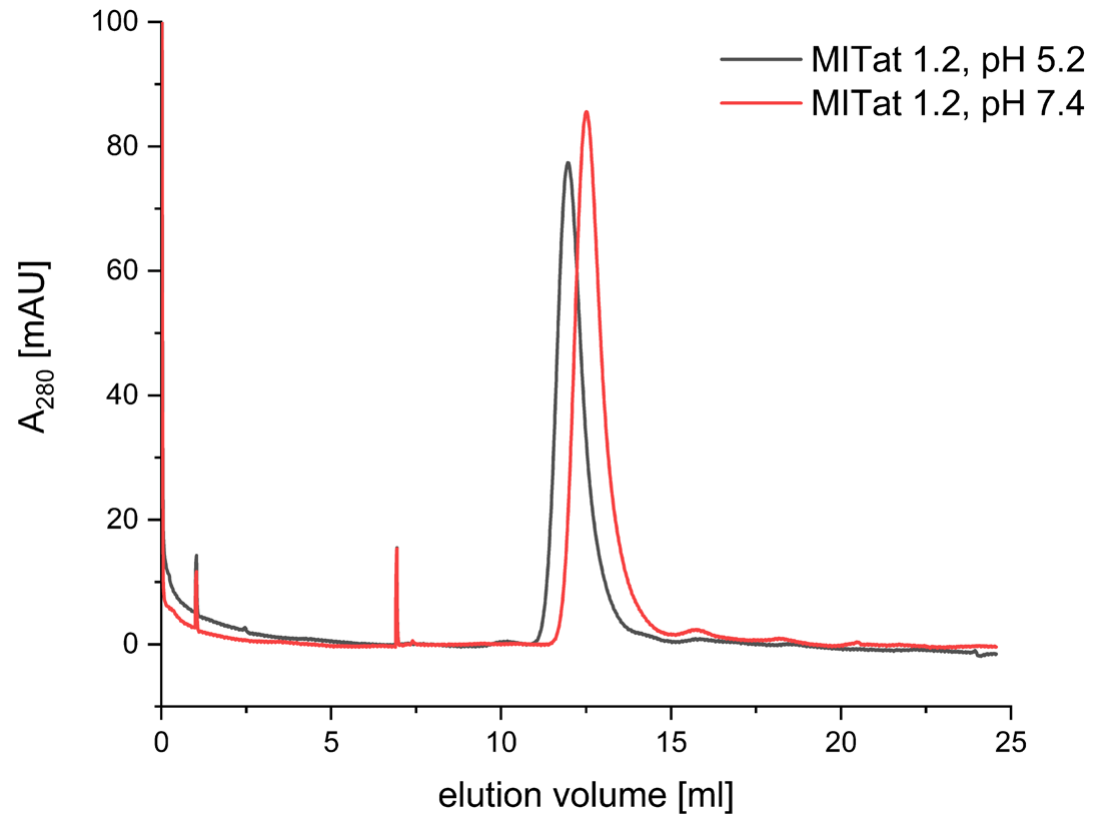
Size exclusion chromatography of MITat1.2 at pH 5.2 and pH 7.4. Shown is the elution volume (x-axis) and the absorption at 280 nm (y-axis). The variant surface glycoprotein (VSG) elution profiles at pH 5.2 (black) and pH 7.4 (red) are compared. MITat1.2 elutes roughly at the same time for both pH values. Minor impurities can be seen at 1 ml and 7 ml elution volume.

Figure 1 shows the elution profile of MITat1.2 for pH 5.2 and pH 7.4. One can see that the two peaks for MITat1.2 are close to each other, with the sVSG eluting slightly earlier at pH 5.2. The maximum of peak of MITat1.2 at pH 5.2 is at 11.96 ml, and for pH 7.4 at 12.50 ml. Thus, the difference between the peaks is 0.54 ml. In addition, small, narrow peaks can be seen at approximately 1 ml and 7 ml elution volume. These can be explained by minor impurities.

### CD measurement

To make statements about the secondary structure of MITat1.2, CD spectroscopy was the method of choice. This is because it is suitable for studying protein structures in solution and under different conditions which in our case was the study of the secondary structure composition of MITat1.2 during acidification within the endosomes following uptake.

**Figure 2.**
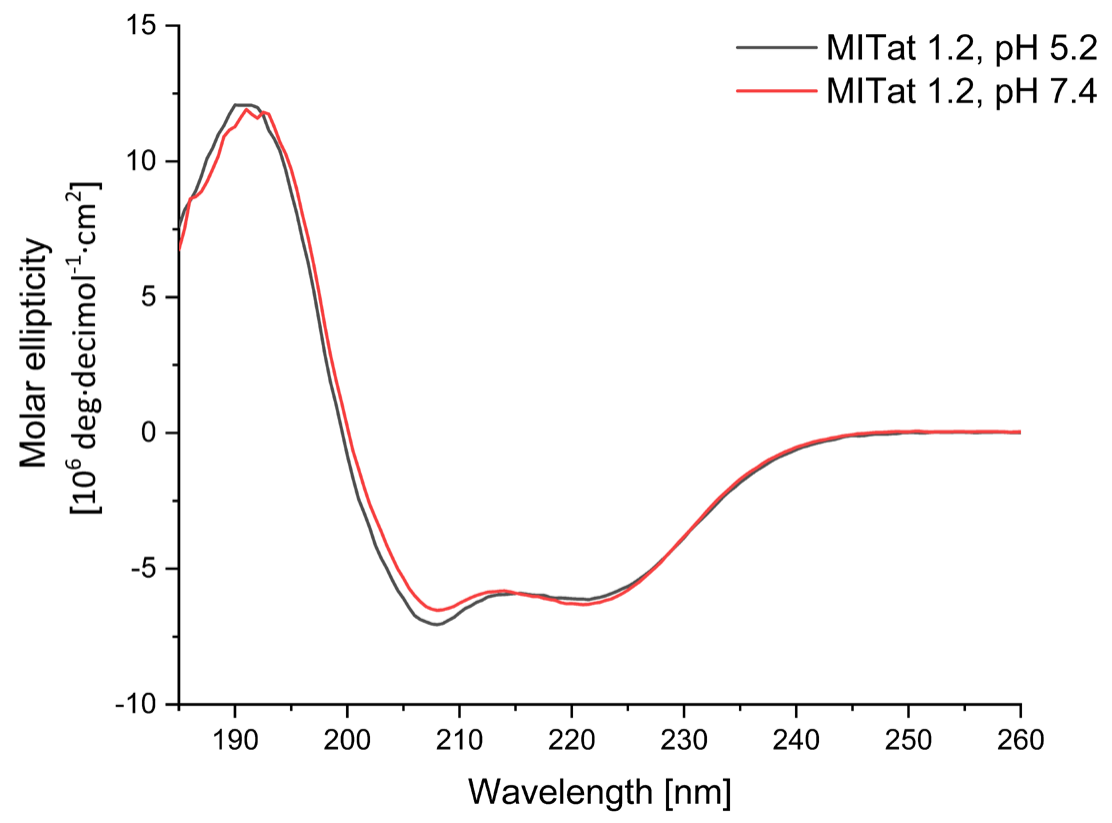
CD spectra of MITat1.2 at pH 5.2 and pH 7.4. Demonstration of spectral shifts observed for each of the different electronic transitions in MITat1.2 at pH of 5.2 (black) and 7.4 (red) as predominantly helical protein. It is notable that not all of the peaks shift in the same direction, nor to the same extent.

Figure 2 shows the two CD spectra for MITat1.2 at pH 5.2 and pH 7.4. The curves for the two different pH values were almost congruent. The signal below 200 nm is exclusively in the positive range. This indicates that the protein is ordered. The two curves show a maximum at 190 nm (pH 5.2) and 191 nm (pH 7.4) and a minimum at 208 nm and 221.5 nm (pH 5.2) and 221 nm (pH 7.4). The ellipticity of the minimum at 208 nm is slightly more negative for pH 5.2. Thus, the shape of the spectra corresponds to that of a protein with a high proportion of α-helices. The analysis of the experimentally acquired data on the DichroWeb online server to determine the content of the various secondary structure elements confirmed this ( Table 1).

**Table 1.** Percentage of secondary structure elements of MITat1.2 at pH 5.2 and pH 7.4. At both pH values MITat1.2 is present as a predominantly helical protein. *

Secondary structure element	MITat1.2 at pH 5.2	MITat1.2 at pH 7.4
α-helix	44 %	44 %
β-sheet	11 %	14 %
β-turn	15 %	12 %
random coil	31 %	29 %

*Since the calculation of the secondary structure elements using the CD data is only an approximation by comparison with a reference set, the sum of the percentages does not result in exactly 100 %.

The calculation was performed by the CDSSTR algorithm with the SMP180 reference set. This combination showed a high agreement of the experimental data with the curve reconstructed by the algorithm. The normalized root mean square deviation (NRMSD) was 0.013 for MITat1.2 at pH 7.4 and 0.007 for MITat1.2 at pH 5.2. Both values indicate that the calculated curves fit the measured data and thus the prediction of the secondary structure fractions is very accurate.

### Molecular Dynamics simulations

Since no experimental model for the intact VSG MITat1.2 protein was available, we used the SAXS based structure model of VSG MITat1.1 to conduct molecular dynamics simulations and subsequent analyses. It is important to note that both VSG structures have a high level of identity, with a similarity of 99.46 %. This ensures that there are no substantial differences between the two structures that could impact the conclusions derived from this study. Based on this comprehensive assessment, we confidently affirm that the results and interpretations presented in this research remain valid.

Crystal structure analysis shows that the N-terminal domain of class A2 VSG proteins forms symmetric homodimers. The C-terminal domain of each VSG monomer links the N-terminal domain of the protein to a GPI-anchor resulting in the VSG protein being anchored to the plasma membrane via two GPI-anchors. Hence the C-termini of both monomers of the homodimer must be pointing towards the plasma membrane to allow anchoring to the membrane and suggests a symmetric or at least close to symmetric positioning of the two C-terminal domains also. While it is technically possible to maintain symmetry in our simulations using all-atom constraint molecular dynamics (MD), this would result in a completely rigid protein structure which would defeat the object of probing for pH based structural differences. We therefore opted not to impose any constraints to the protein structure, which would affect the conformational changes in the VSG molecule, but we acknowledge that the resulting asymmetry of the VSG homodimer is most likely not compatible with the constraints the VSG encounters while anchored to the membrane. We believe that this approach is the best way to capture the essential features of the VSG protein's dynamics.

Molecular dynamics simulations were utilized to determine the conformational changes in MITat1.1 at different pH levels. Secondary structure studies were conducted on optimal conformations of MITat1.1 found by PCA from the molecular dynamics trajectories.

Table 2 shows calculated secondary structure elements for MITat1.1 at pH 5.2 and 7.4 in comparison to experimental data ^30^. MITat1.1 has a slightly more ordered structure in an acidic medium which is indicated by a lower amount of random coil secondary structure elements. One can also note a slight general decrease in α-helix and β-sheet structure elements in optimized protein geometries in comparison to reference NMR and crystal structure based values ^13, 42^. The protein in the simulation is dissolved in the explicit water while X-Ray data is from crystallized protein. Therefore, a slight decrease in the structural order of a protein may be neglected.

**Table 2.** Percentage of secondary structure elements (α-helix, β-sheet and random coil) of MITat1.1 at pH 5.2 and pH 7.4 predicted from MD data in comparison to the secondary structure found in the literature. Calculated values are mainly in the accordance with the reference. MD - molecular dynamics.

Secondary structure element	MITat1.1 at pH 5.2	MITat1.1 at pH 7.4	Reference ^13, 42^
α-helix	41 %	39.4 %	42,8 %
β-sheet	0.9 %	0.6 %	3.0 %
random coil	57.1 %	60 %	54,2 %

**Figure 3.**
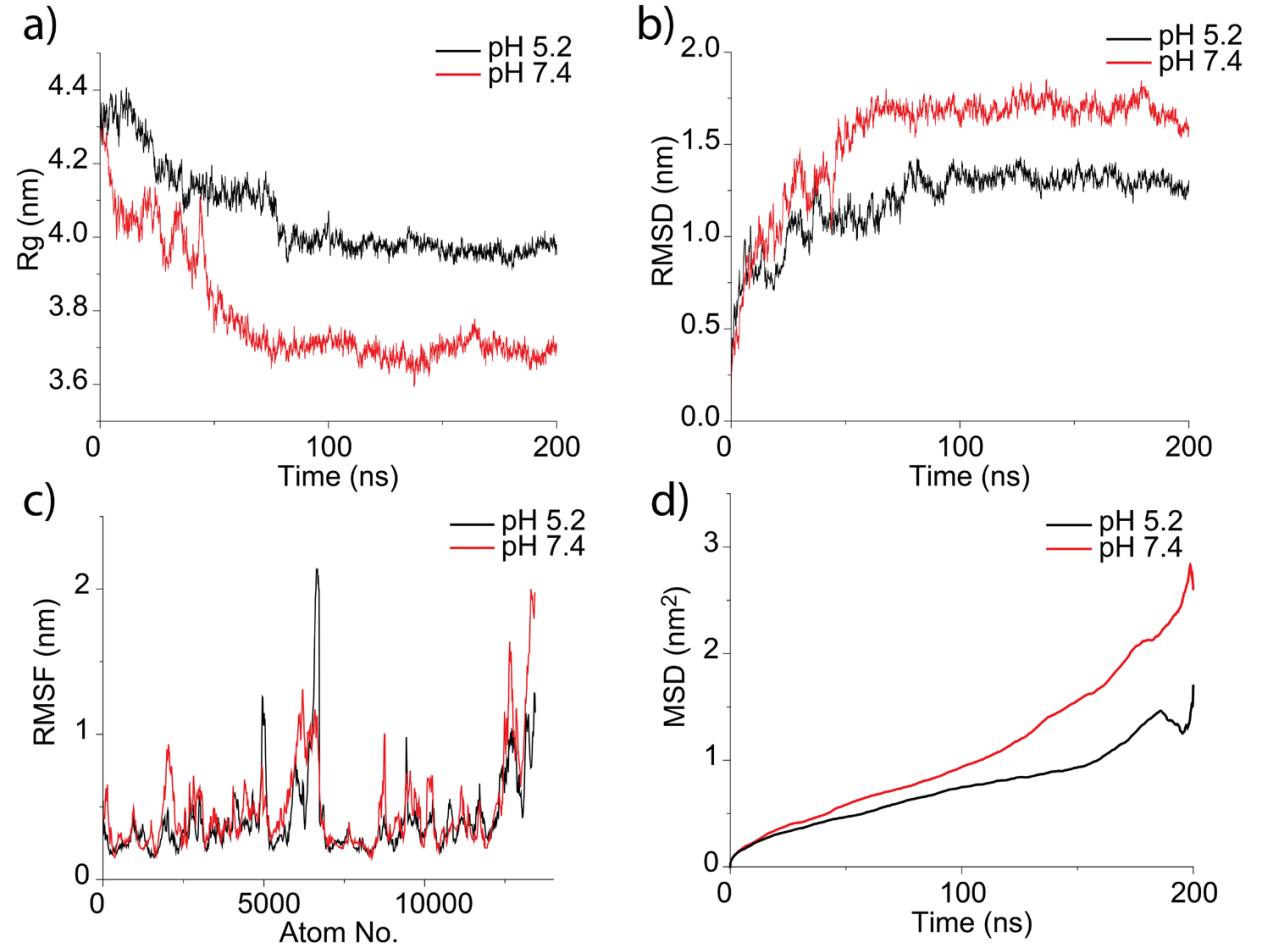
Data obtained from molecular dynamics simulations of MITat1.1 at pH 5.2 (black) and pH 7.4 (red): ( **a**) gyration radius (Rg) of the protein, ( **b**) root mean square deviation (RMSD) of the protein, ( **c**) root mean square fluctuation (RMSF) of atoms in the protein, ( **d**) mean square deviation (MSD) of the protein. The Rg values suggest bigger size of the protein at pH 5.2.

In order to gain insight into the conformational change of MITat1.1 gyration radius ( Figure 3 (a)), root mean square deviation ( Figure 3 (b)), root mean square fluctuation ( Figure 3 (c)), mean square deviation ( Figure 3 (d)) were calculated. RMSF shows that the protein at pH 5.2 is most flexible in the middle of the structure, while the protein at pH 7.4 has a more flexible terminus. The data obtained from the gyration radius graph provided evidence that MITat1.1 at pH 5.2 has a bigger linear size than MITat1.1 at pH 7.4 which is consistent with the experimental data from SEC. The higher number of protonated residues at acidic conditions may lead to a more intensive repulsion between these residues, that is to a bigger gyration radius. MITat1.1 has a bigger deviation from the initial structure at pH 7.4 which may be explained by a higher content of disordered random coil secondary structure element at these conditions.

**Figure 4.**
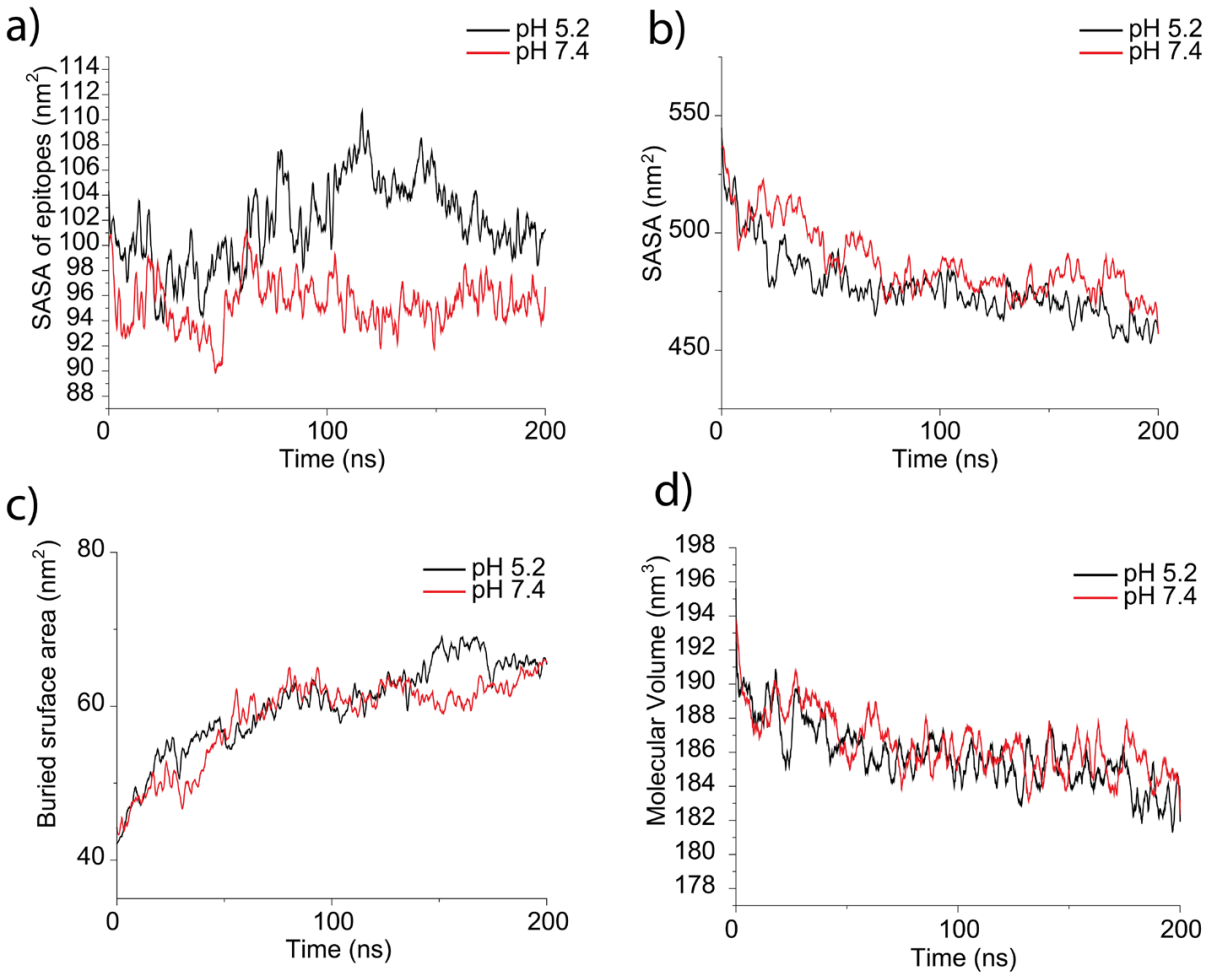
Data obtained from molecular dynamics simulations of variant surface glycoprotein (VSG) dimer at pH 5.2 (black) and pH 7.4 (red): ( **a**) solvent accessible surface area of epitopes (SASA of epitopes), ( **b**) solvent -accessible surface area of the protein (SASA), ( **c**) buried surface area (BSA), and ( **d**) molecular volume of the protein. The data suggests more accessible epitopes at pH 5.2.

**Figure 5.**
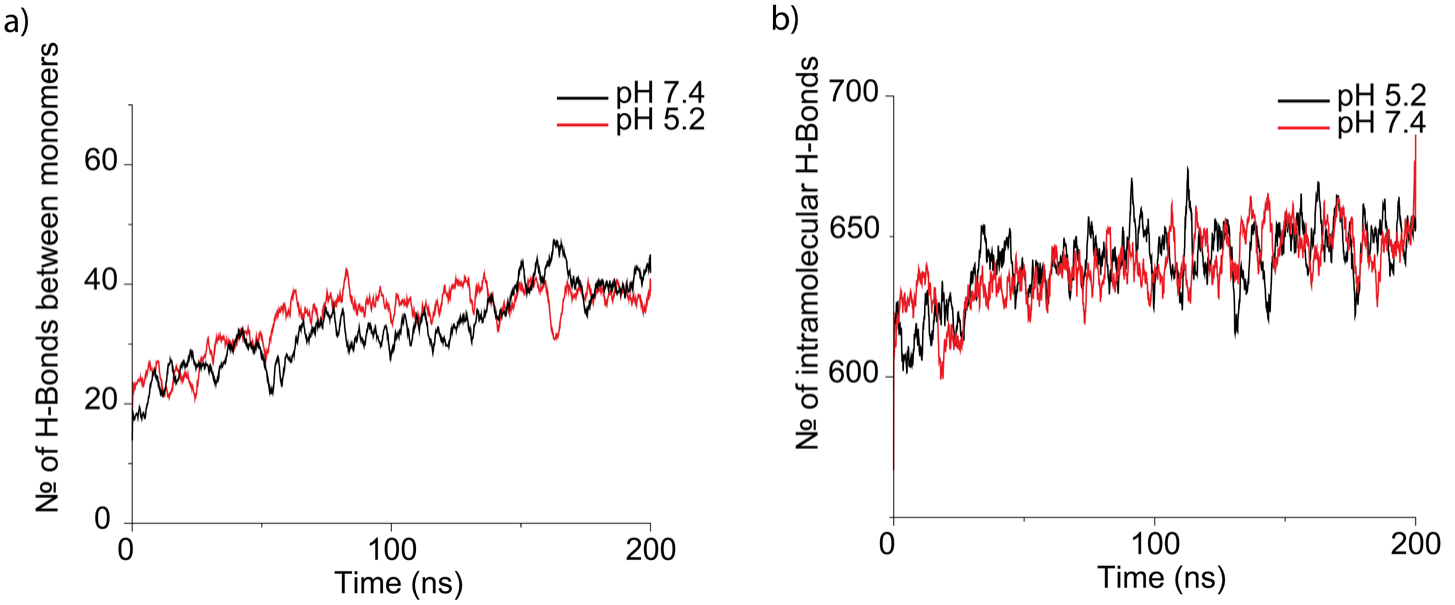
Data obtained from molecular dynamics simulations of variant surface glycoprotein (VSG) dimer at pH 5.2 (black) and pH 7.4 (red). ( **a**) The number of hydrogen bonds between monomers, ( **b**) the number of intramolecular hydrogen bonds. There is no clear distinction in the number of formed hydrogen bonds between the protein at pH 5.2 and pH 7.4.

Figure 4 shows other calculated parameters: solvent-accessible surface area of epitopes ( Figure 4 (a)), solvent-accessible surface area ( Figure 4 (b)), buried surface area ( Figure 4 (c)) and molecular volume Figure 4 (d)). The calculated number of intramolecular and intermolecular hydrogen is shown in Figure 5. Table 3 shows averaged values for the last 100 ns. From Table 3 and Table 4 one can conclude that epitopes are more accessible for solvent (and therefore for antibodies) at pH 5.2. Such phenomena seem to be connected to the size of a protein molecule – with the increase in size, the accessibility of epitopes increases. Moreover, it seems that the buried surface area for protein at pH 5.2 is slightly bigger as well as the solvent-accessible surface area at the same pH. Molecular volume and numbers of intermolecular and intermolecular hydrogen bonds do not drastically differ between MITat1.1 at pH 5.2 and a 7.4.

**Table 3.** Averaged data from the last 100 ns of simulation for VSG dimer at pH 5.2 and pH 7.4. VSG - variant surface glycoprotein, SASA – Solvent accessible surface area, BSA – Buried surface area, Rg – Gyration radius. Rg and SASA of epitopes values suggest the larger conformation at pH 5.2 and therefore the larger degree of accessibility of epitopes at pH 5.2.

Parameter	MITat1.1 at pH 5.2	MITat1.1 at pH 7.4
SASA, nm ^2^	469.0±8.1	478.0±7.8
SASA of epitopes, nm ^2^	104.0±2.6	95.0±1.3
BSA, nm ^2^	64.0±3.0	62.0±1.9
Molecular volume, nm ^3^	185.0±2.2	186.0±2.1
Number of intermolecular H-bonds	37.0±4.5	38.0±2.2
Number of intramolecular H-bonds	646.0±10.0	647.0±8.5
Rg, nm	3.97±0.02	3.69±0.03

**Table 4.** Averaged data from the last 100 ns of simulation for VSG dimer at pH 5.2 and pH 7.4. VSG - variant surface glycoprotein, SASA – Solvent accessible surface area, BSA – Buried surface area, Rg – Gyration radius. ∆G - The free Gibbs energy of binding between two monomers in the dimer is lower at pH 5.2.

Parameter	MITat1.1 at pH 5.2	MITat1.1 at pH 7.4
SASA, nm ^2^	451	459
SASA of epitopes, nm ^2^	102	96
BSA, nm ^2^	66	65
Molecular volume, nm ^3^	182	183
Number of intermolecular H-bonds	42	39
Number of intramolecular H-bonds	653	686
Rg, nm	3.95	3.71
∆G, kcal/mol	-353.37	-324.07
TS, REU	-370.55	-349.59

**Figure 6.**
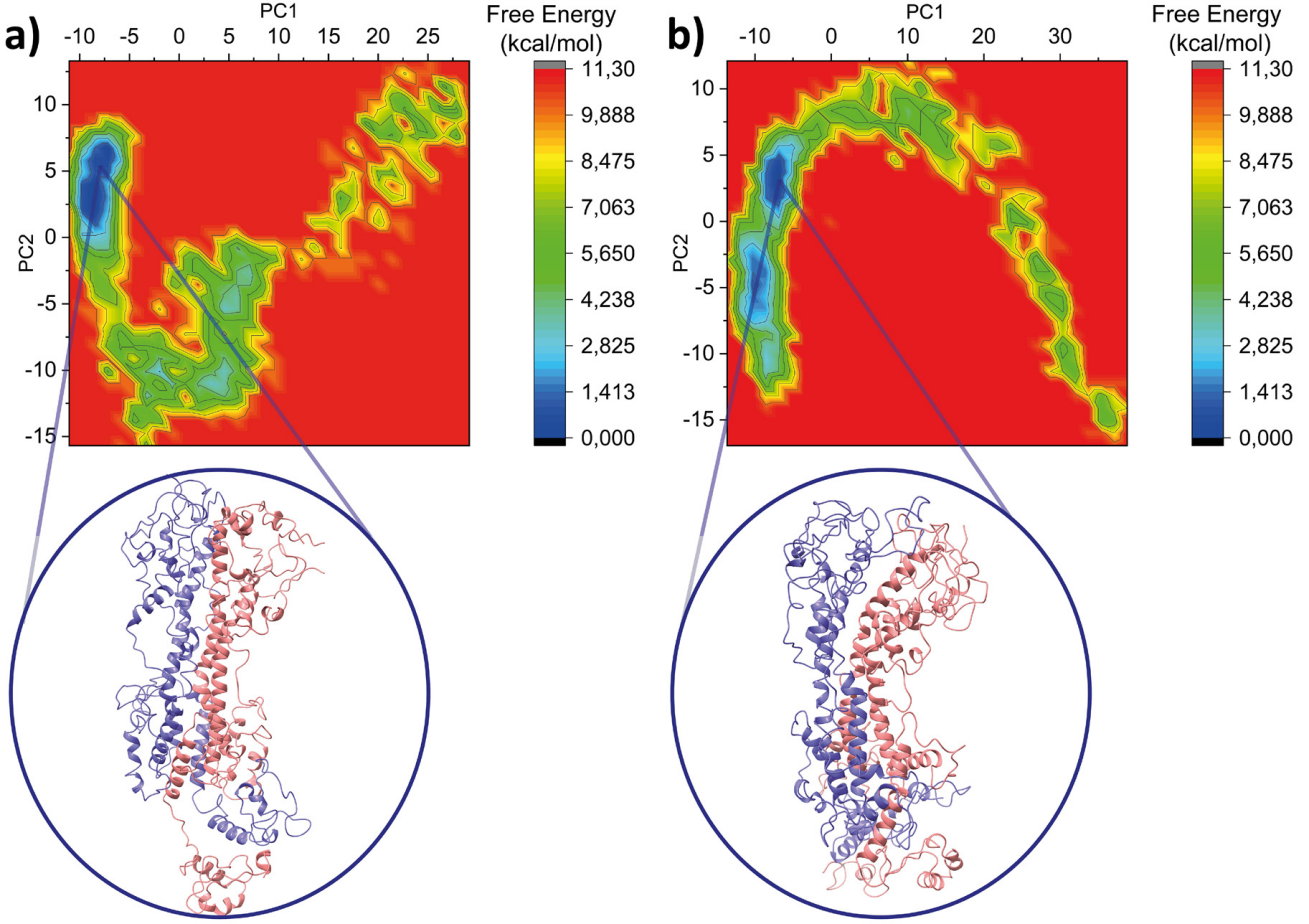
Free Energy Landscape for MITat1.1 at pH 5.2 (a) and pH 7.4 (b) presents the distribution of free energy states of MITat1.1 in relation to different protein conformations. The 2D plot depicts different protein conformations, with energy minima indicating stable states and maxima suggesting less stable ones. The landscape highlights potential structural transitions and conformational changes. Darker regions signify lower free energy, representing more stable conformations.

**Figure 7.**
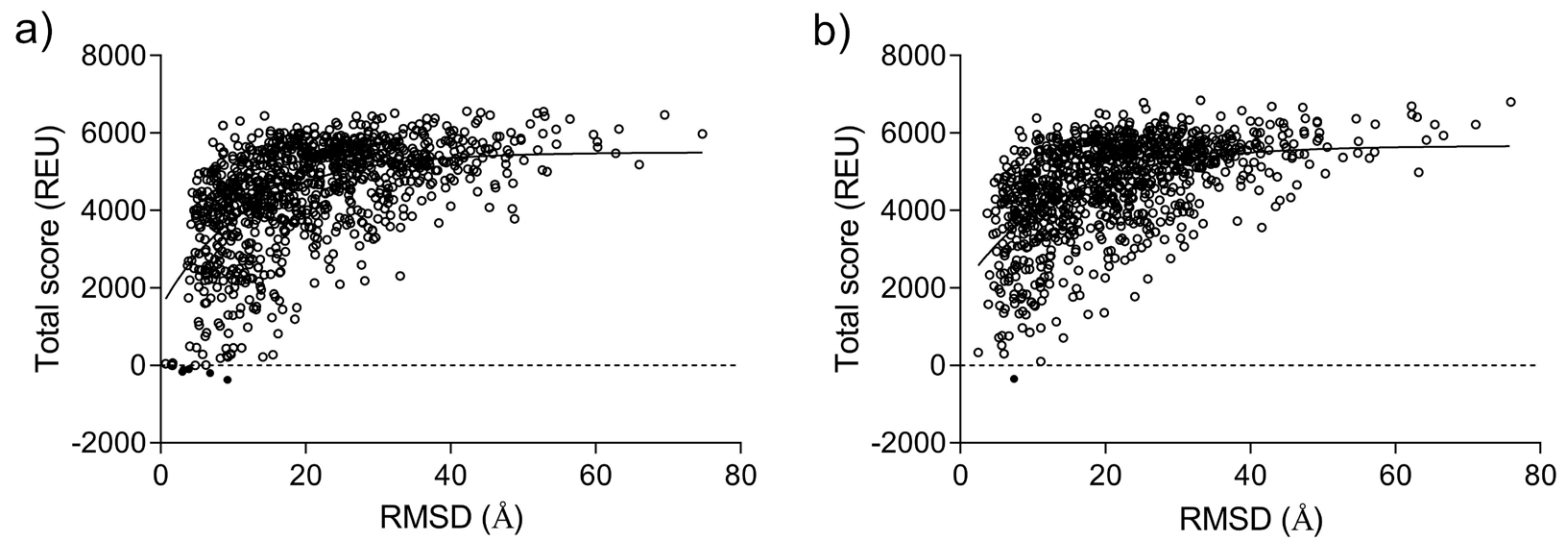
ROSETTA energy score versus root-mean-square-deviation (RMSD) plots for full-length variant surface glycoprotein (VSG) models at pH 5.2 (a) and pH 7.4. (b) using the Monte Carlo algorithm. The energy threshold is depicted as a dashed line. The REU is abbreviated for the Relative Energy Unit.

Figure 6 shows the free energy landscape for MITat1.1 at pH 5.2 (a) and pH 7.4 (b). Table 4 shows the main calculated parameters with the addition of free Gibbs binding energy of optimized geometries of MITat1.1 at pH 5.2 and pH 7.4. The lower binding energy of MIT at1.1 at pH 5.2 is consistent with a slightly bigger buried surface area and indicates better binding of monomers at acidic pH. Additionally, similar results were obtained from the Monte-Carlo simulation of MITat1.1 at pH 5.2 and pH 7.4, where the monomers were more attached to each other at low than at high pH ( Figure 7 (a, b), Table 4).

**Figure 8.**
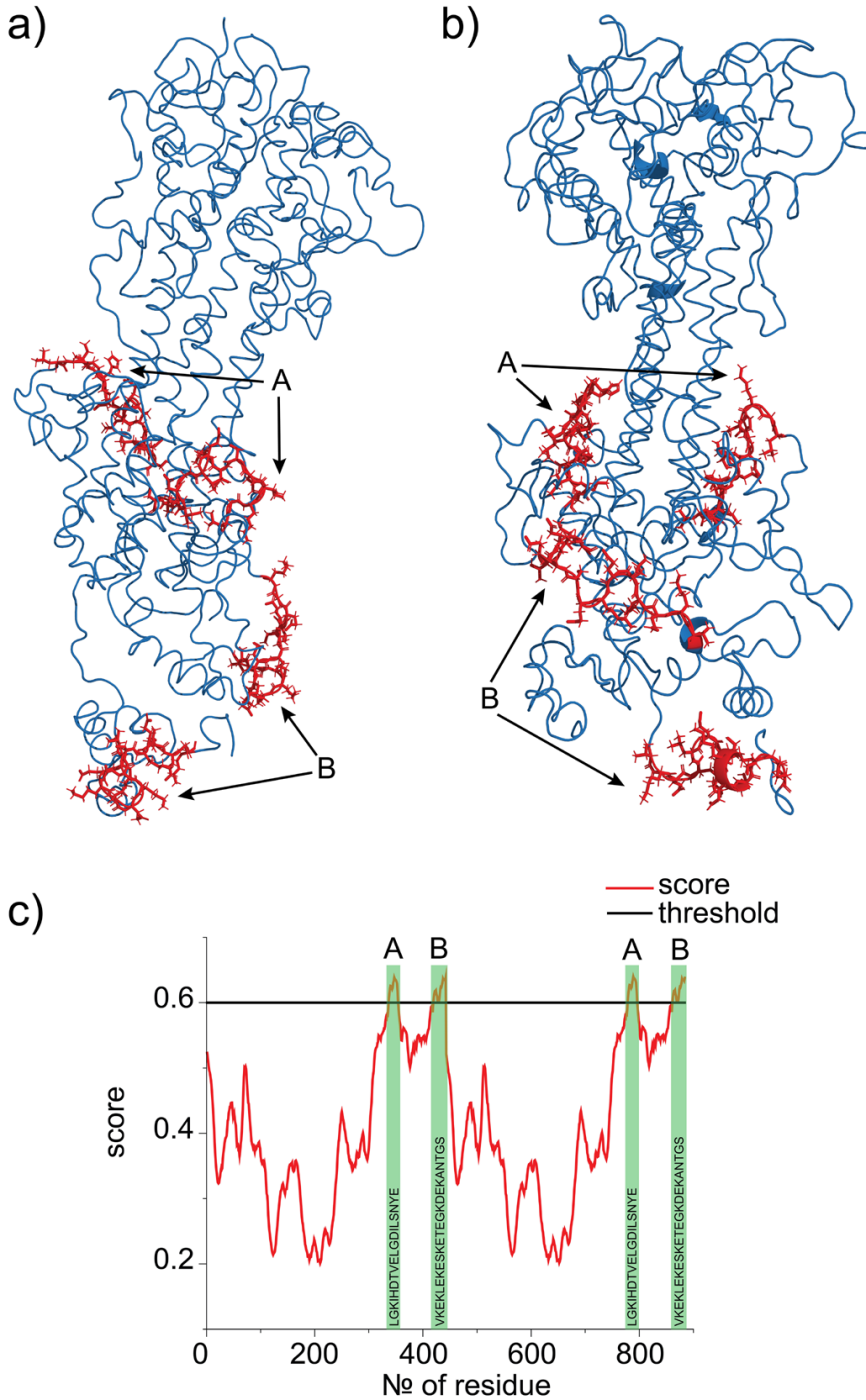
Optimized geometries highlighted by red epitope sequences for variant surface glycoprotein (VSG) Dimer. A comparison of geometries at pH 5.2 ( **a**) and pH 7.4 ( **b**) is shown. Graph ( **c**) represents the results from EpiDope analysis. The black line represents a threshold for the amino acid sequence which is to be considered as epitope. The epitopes are placed at the end and in the middle of the protein. These are also the most flexible parts of the protein.

Figure 8 shows the epitope sequences and results from the EpiDope program. It indicates that most conformational changes of epitopes are happening in the middle and at the end of the protein which are also the most flexible parts according to the RMSF data. This, in turn, indicates that epitope regions of the protein are more responsive to any external changes and therefore can be controlled by precise changes in the solution.

## Discussion

Host antibodies are thought to be efficiently removed during the endosomal passage of VSG ^IgG^ in trypanosomes, and this happens even without a larger pH-dependent conformational change. That the VSG is comparatively stable at different pH relies on the following evidence: Direct measurement of structural stability of VSG (construct MITat1.2) both in SEC and CD measurements as well as of MITat1.1 in molecular dynamics, *in silico* structure analysis, and energy calculations. We show that there is only a minor effect on VSG structure by pH changes occurring e.g., in the endosomal pathway.

**SEC measurements**: MITat1.2 is present as a homodimer at pH 5.2. If it were present as a monomer, this would lead to a shift of the elution maximum to a later point in time, since molecules with a small hydrodynamic diameter are retained longer in SEC. However, the results show the opposite: MITat1.2 elutes slightly earlier at pH 5.2. Consequently, the hydrodynamic diameter seems to be slightly larger. However, the difference is too small to assume that protein association is greater than dimer formation. Comparable studies showed more divergent peaks in SEC for different aggregates of various proteins ^43– 45^.

It would be conceivable that the difference in the peaks is due to the use of two different buffers. The flow properties of HEPES and sodium acetate buffers could be different, so the elution maxima - regardless of pH - are not comparable and should appear to be contemporaneous. However, this does not seem to be the case, since the remaining impurities in the sample elute at the same time in both measurements. The difference can therefore not be explained by the flow properties of the buffers. In conclusion, the difference in the two elution peaks of MITat1.2 at pH 7.4 and pH 5.2 in SEC is too small to attribute it to protein association other than dimer formation.

**CD observations**: With the CD measurement performed and subsequent analysis on DichroWeb, a proportion of 44 % could be calculated for both pH values. This is in agreement with previous experiments. For MITat1.2, an α-helix fraction of 47 % for the N-terminal domain and 24 % for the C-terminal domain was already determined by crystal structure analysis and NMR spectroscopy analysis, respectively ^13, 42^. This results in 43 % α-helix fraction for the whole protein. The proportions of the other secondary structure elements are also very similar and show hardly any differences. Thus, the secondary structure of VSG MITat1.2 is stable even at an acidic pH of 5.2.

However, the SEC result could be indicative of a smaller tertiary structure change. For enzymes, a small change in the three-dimensional structure of the function-bearing part is often sufficient to cause a large change in the activity of the enzyme. A similar situation can occur with the epitope of the VSG. If a structural change occurs here, the binding pattern could change, and the specific antibody could no longer bind. In fact, the epitope-bearing part of the VSG at the N-terminal end of the protein is a site where many loops and random coils occur. Therefore, a change at this site might not be particularly noticeable in CD spectroscopy but could still lead to a relevant change in function.

On the other hand, it is clear from the molecular dynamics simulations that the dimer size of MITat1.1 is slightly bigger at pH 5.2 than at pH 7.4 in agreement with the SEC data. Due to the higher SASA value of epitopes predicted at pH 5.2 ( Figure 4 (b)), it can be speculated that these epitopes are more exposed to the solvent at low pH making them more accessible to the neutralizing antibodies. Additionally, moderate conformational changes (ΔRMSD = 0.35 nm) in VSG were detected between the dimers simulated at different pH based on the published reference data ^46^. According to Marsh and co-authors, solvent-accessible surface area ( *A*rel) between 1.1 and 1.2 can be used as an indicator of moderate conformational change (>0.2 nm RMSD). On the contrary, the proteins with *A*rel values >1.2 (>0.5 nm RMSD) and <1 (<0.2 nm RMSD) are more likely considered to undergo significant and small conformational changes ^46^.

Taking into consideration these results, one could also speculate that this conformational shift might be responsible at some point for the high exposure of epitopes to a solvent, antibodies etc., at low pH and *vice versa*. This can be explained by the fact that protein structures at this pH tend to be more ‘open’ or 'unfolded', where the ionic attraction disappears unfolding the protein shape ^47^. Moreover, the increase in the monomer-monomer interaction interface (BSA) might be also associated with this 'open' state of the molecule followed by the affinity elevation in a dimer ( Table 4, Figure 7).

It is a well-established strategy to simulate soluble proteins and protein-ligand complexes for at least 50–100 ns using standard force fields to measure important structural and interaction parameters ^48, 49^. In our study, sufficient simulation time (200 ns) was applied to observe the protein conformational changes, which could also be used to investigate more complex transmembrane proteins. In particular, the same time-scale protocol was already implemented in various studies to analyse transmembrane dopamine D1/D2 receptors or some multidrug resistance proteins ^50, 51^.

The acidic pH alone cannot be considered the primary factor for antibody dissociation from VSG ^IgG^. An alternative explanation could be the presence of specific proteases that selectively degrade host antibodies without affecting the VSGs. Such a mechanism has been suggested for the trematode *Echinostoma caproni,* where antibody trapping and subsequent degradation aid in evading the host immune response ^52^. The fact that African trypanosomes can infect a wide range of hosts complicates the problem. Not all mammals express the same antibody species. For instance, in bovines, the presence of very long CDR-H3 structures gives rise to an additional minidomain on antibodies. This minidomain extends from the antibody surface, exhibiting sequence diversity and varying disulfide patterns ^53^. Therefore, it is highly unlikely that broad-specificity proteases targeting antibodies, but sparing VSGs exist in trypanosomes.

Currently, we favour an alternative solution that is (1) simple, (2) independent of the host's antibody species, and (3) does not rely on any biochemical mechanisms. Formally, the release of VSG from VSG ^IgG^ has not been demonstrated thus far. Additionally, it is important to note that antibody clearance is only operational during an early immune response, as the hydrodynamic drag force must be able to engage the VSG ^IgG^. Consequently, the presence of antibodies on the cell surface must be limited. This raises the question whether the parasite might not simply be able to compensate for the loss of VSG complexed with antibodies. We are currently in the process of testing this hypothesis.

## Ethics and consent

Ethical approval and consent were not required.

## Data Availability

Figshare: The release of host-derived antibodies bound to the variant surface glycoprotein (VSG) of Trypanosoma brucei cannot be explained by pH-dependent conformational changes of the VSG dimer. https://doi.org/10.6084/m9.figshare.24716316 ^20^. Data are available under the terms of the Creative Commons Attribution 4.0 International license (CC-BY 4.0).
