# Supplementary material for: The release of host-derived antibodies bound to the variant surface glycoprotein (VSG) of Trypanosoma brucei cannot be explained by pH-dependent conformational changes of the VSG dimer
Source: Open Res Eur. 2024 Apr 24;4:87. [Version 1] doi: 10.12688/openreseurope.16783.1 (PMC11187536; doi:10.12688/openreseurope.16783.1)
Supplement: Peer review history

# Reviewer report: Reviewer response for version 1

**DOI:** 10.21956/openreseurope.18130.r41010

**Published:** 2024-06-19

**Authors:** Monica R. Mugnier

**Affiliations:** 1 Johns Hopkins Bloomberg School of Public Health, Baltimore, USA

In this study, Eirich, et al investigate the intriguing question of how T. brucei sorts VSG from antibody in the endosome, so that endocytosed antibody can be routed to the lysosome and degraded. One hypothesis is that the lower pH in the endosome causes structural changes to the VSG that facilitate dissociation of the VSG from bound antibody. The authors perform rigorous in vitro and in silico analyses to investigate how VSG structure might change with a change in pH. Their analyses show that protein changes at low pH are minimal and suggest that this indicates pH is unlikely to be the primary mechanism driving dissociation of VSG from antibody in the endosome. Overall, the manuscript is exceptionally clear and very accessible even for a reader without a strong background structural biology and biochemistry. I appreciated how clearly the authors articulated their rationale for most experimental choices they made in the study. I only have a few minor suggestions about the presentation and interpretation that may help improve the clarity of the paper.

 My primary concern is in the interpretation of the data. While I agree that the changes are minimal, there are changes. It remains possible these small changes could facilitate dissociation of the antibody-VSG complex (which is in fact mentioned at some points in the manuscript). Therefore, I would suggest a more tentative interpretation in the discussion section--I think it is fair to say one model is favored, but, given that there are some changes at pH 5.2, it is possible that those small changes are sufficient. I don't think this more nuanced presentation would undermine the study, and instead would leave readers with a more clear understanding of what could be going on.

 I was also a bit confused about how to interpret the epitope accessibility analysis. At pH 5.2, epitopes are largely more accessible. Do any parts of the protein become less accessible? If this is the case, then it still remains possible that certain epitopes do change sufficiently to interfere with antibody-VSG binding. It may also be worth acknowledging the limitations of epitope prediction (especially in the context of VSG, where very little data exist) when discussing these data. Finally, in figure 4A, there is an analysis of solvent accessible surface area of epitopes. Are these the same epitopes described in Figure 8? I assume so, but this wasn't completely clear to me from the text.

 I also wondered whether some other factors could influence these pH effects. Can the concentration of VSG in the endosome be estimated, and would this affect folding? How does the concentration in these experiments compare? Is the presence of bound antibody likely to affect/restrict the potential spectrum of changes in folding at low pH? I do not think it is essential to experimentally test these variables, but if they are likely to have any influence it could make sense to mention them.

 Finally, I would suggest the authors adjust their abstract/introduction slightly, given the hypothesis they put forth in the final sentences of the paper. In the abstract they state "Bound antibodies are separated in the endosome from the VSG and destroyed in the lysosome." The introduction states, "VSGIgG is rapidly internalized through clathrin-mediated endocytosis. Within the endosomes, the antibodies are separated from VSGIgG and sorted through late endosomes to a single lysosome, where they are destroyed. In contrast, the VSG is efficiently directed to recycling endosomes via the flagellar pocket and subsequently returned to the cell surface." I knew this was the accepted understanding in the field, but their hypothesis at the end of the paper--that perhaps VSG is just degraded, and this loss is compensated for by the parasite--suggests there isn't evidence for the idea that VSG is recycled back to the surface! If this is the case, I would recommend making these statements in the abstract a little more tentative. If they are planning to counter this dogma (an exciting possibility) I think it makes sense to avoid perpetuating it for now.

Is the study design appropriate and does the work have academic merit?

Yes

Is the work clearly and accurately presented and does it cite the current literature?

Yes

If applicable, is the statistical analysis and its interpretation appropriate?

Yes

Are all the source data underlying the results available to ensure full reproducibility?

Yes

Are the conclusions drawn adequately supported by the results?

Partly

Are sufficient details of methods and analysis provided to allow replication by others?

Yes

Reviewer Expertise:

T. brucei antigenic variation and host-pathogen interactions

I confirm that I have read this submission and believe that I have an appropriate level of expertise to confirm that it is of an acceptable scientific standard.

# Reviewer report: Reviewer response for version 1

**DOI:** 10.21956/openreseurope.18130.r41008

**Published:** 2024-06-19

**Authors:** Rajender Kumar

**Affiliations:** 1 ICAR-National Research Centre on Equines, Haryana, India

**General Comments**

 African trypanosomiasis caused by *Trypanosoma brucei gambiense* and *Trypanosoma brucei* *rhodesiense *are responsible for a chronic and an acute form of sleeping sickness, respectively. These *Trypanosoma* spp. are extracellular protozoa and survive in the hostile environment of the mammalian bloodstream. These parasites are continuously targeted by the host's innate and specific host defense mechanisms. They have developed potent evasion strategies for their survival. Their primary defense mechanism involves changing their surface coat, which is composed of a variant surface glycoprotein (VSG), through stochastic activation of different VSG genes. The VSG envelops the entire trypanosome cell and come in direct contact with host antibodies. The parasite clears VSG bound antibodies by endocytosis through flagellar pocket, a small invagination of the plasma membrane. The process of dissociation of anti-VSG-antibodies in endosomes is yet to be explained.

  In the present manuscript, the authors have attempted to demonstrate pH-independent dissociation of anti-VSG-antibodies in endosomes using various methodologies viz, size-exclusion chromatography, circular dichroism spectroscopy, molecular dynamics simulations, etc. The findings are novel and well explained.

**Specific comments:**

 Here are some minor comments and suggestions which need to be incorporated:

 Introduction:

 Slightly lengthy. Text of paragraph 2 and 3 may be merged and reduced.

 Introduction section last paragraph: Only one sentence is enough “In this study, we present the results of biochemical experiments and numerical simulations aimed attesting this hypothesis”. No need to explain results in the introduction part.

 Methods:

 Paragraph-  Call harvest, Line 1: GPI-PLC activity. (expand the abbreviated form)

 Paragraph  Call harvest, Line 6: TLCK (expand the abbreviated form)

 Paragraph “Preparation for size-exclusion chromatography and circular dichroism measurements” may be removed. And text may be shifted to next paragraphs - size-exclusion chromatography, circular dichroism measurements.

 Paragraph -Size-exclusion chromatography:

 “SEC of MITat1.2 at pH 7.4 was performed on an ÄKTA pure using a Superdex 200 Increase 10/300 GL column and 50 mM HEPES buffer (pH 7.4) with 150 mM NaCl as the running buffer”. Text to be rewritten with more clarity.

 Also, at various places in methodology section, abbreviated forms are written. Procedures/ techniques used need slight more explanation for better understanding.

 Results:

 Interpretations of the Figures and Tables presented in the results section should be there in the text of results for better understanding of the findings.

 In ‘Molecular dynamics simulations’ paragraph of results section authors have explained  methodology in this section.  Explain only findings at this place.

 There is lot of text explained in the Table 2 caption. Move the text in results section.

 Discussion:

 Needs to concise. Lot of irrelevant text. Findings need to be discussed in a focused manner

 Last paragraph text should conclude the findings and way forward for future studies.

**The manuscript contains valuable and significant novel information for the scientific community working in the area. The manuscript may be considered for indexing after revision as indicated above.**

Is the study design appropriate and does the work have academic merit?

Yes

Is the work clearly and accurately presented and does it cite the current literature?

Yes

If applicable, is the statistical analysis and its interpretation appropriate?

Yes

Are all the source data underlying the results available to ensure full reproducibility?

Yes

Are the conclusions drawn adequately supported by the results?

Yes

Are sufficient details of methods and analysis provided to allow replication by others?

Yes

Reviewer Expertise:

Molecular diagnostics and newer target specific  drug development against trypanosomatids.

I confirm that I have read this submission and believe that I have an appropriate level of expertise to confirm that it is of an acceptable scientific standard.
